# Vaginal Microbiota and Proinflammatory Status in Patients with Polycystic Ovary Syndrome: An Exploratory Study

**DOI:** 10.3390/jcm13082278

**Published:** 2024-04-15

**Authors:** María Elena Espinosa, Angélica Melo, Marion Leon, Estefanía Bautista-Valarezo, Fabiola Zambrano, Pamela Uribe, Anita Bravo, Anja Taubert, Carlos Hermosilla, Virginia Iturrieta, Raul Sánchez

**Affiliations:** 1 Ph.D. Program in Medical Sciences, Faculty of Medicine, Universidad of La Frontera, Temuco 4780000, Chile; meespinosax@utpl.edu.ec; 2 Department of Health Sciences, Faculty of Health Sciences, Universidad Técnica Particular de Loja, UTPL, San Cayetano Alto s/n, Loja 1101608, Ecuador; mebautista@utpl.edu.ec; 3 Center of Excellence in Translational Medicine-Scientific and Technological Bioresource (CEMT-BIOREN), Temuco 4780000, Chile; angelica.melo@ufrontera.cl (A.M.); m.leon06@ufromail.cl (M.L.); fabiola.zambrano@ufrontera.cl (F.Z.); pamela.uribe@ufrontera.cl (P.U.); a.bravo02@ufromail.cl (A.B.); v.iturrieta01@ufrontera.cl (V.I.); 4 Department of Preclinical Sciences, Faculty of Medicine, Universidad of La Frontera, Temuco 4780000, Chile; 5 Department of Internal Medicine, Faculty of Medicine, Universidad of La Frontera, Temuco 4780000, Chile; 6 Institute of Parasitology, Justus Liebig University Giessen, 35392 Giessen, Germany; anja.taubert@vetmed.uni-giessen.de (A.T.); carlos.r.hermosilla@vetmed.uni-giessen.de (C.H.)

**Keywords:** polycystic ovary syndrome, phenotypes, vaginal inflammatory reaction, vaginal microbiota, neutrophil extracellular traps (NETs)

## Abstract

**Background/Purpose**: Polycystic ovary syndrome (PCOS) is an endocrine-metabolic disease most common in patients of childbearing age. This pathology is associated with clinical, metabolic, and reproductive complications. We evaluated the diversity of the vaginal microbiota (VM), the vaginal inflammatory reaction (VIR), the proinflammatory state, and the activation of polymorphonuclear neutrophils (PMN) with the production of neutrophil extracellular traps (NETs). **Methods**: Thirty-three patients who attended a consultation at the Hospital UTPL-Santa Inés, Loja, Ecuador, from May to August 2023 who were diagnosed with PCOS participated in this study. Blood samples, vaginal discharge, and a survey were obtained. **Results**: A high number of patients, 23/33 (69.7%), presented altered microbiota in clinical variables associated with PCOS phenotypes A and B, sexual partners (>2), and oligomenorrhoea. A significant statistical association was only observed for sexually transmitted infections at sampling (*p* = 0.023) and insulin (*p* = 0.002). All eight cases studied with VIR had PMN/NETotic activity. A high frequency of proinflammatory states was observed in all vaginal microbiota states. **Conclusions**: These results suggest that the PCOS could trigger a proinflammatory state in the vaginal epithelium independently of the state of the vaginal microbiota. Furthermore, the presence of NETs observed in the cases studied could decrease fertility in these PCOS patients.

## 1. Introduction

Polycystic ovary syndrome (PCOS) is a neuroendocrine, metabolic, and reproductive disorder that can affect up to 20% of women in their reproductive age. It is characterized by oligo-anovulation, clinical and/or biochemical hyperandrogenism, and ultrasonography with diagnosis of polycystic ovaries [1]. Applying the recommendations of the 2019 international evidence-based guideline for diagnosing PCOS, the diagnosis of the different phenotypes of PCOS (A, B, C, and D) can be made, allowing its categorization and type of treatment [2,3]. Its etiopathogenesis is complex, multifactorial, and heterogeneous in its presentation, including the interaction of genetic, epigenetic, and environmental factors [4]. There is a close association between microbial dysbiosis and pathological changes in PCOS [3].

In this association, the release of proinflammatory and inflammatory factors, the primary mechanism driven by altered microbiota, could be associated with the clinical characteristics of polycystic ovary syndrome, predominantly obesity, insulin resistance, and vitamin D deficiency in the different phenotypes of patients with PCOS [5,6]. Likewise, there is an imbalance in the vaginal microbiota, generated mainly by irregular menses and hormonal changes associated with hyperandrogenism and hyperinsulinemia with anovulation and oligo- or amenorrhea. This maintains an estrogenic tone not counteracted by postovulatory progesterone, so that estrogen stimulates the accumulation of glycogen in the cells of the vaginal epithelium. This glycogen is used by Lactobacillus within vagina to produce lactic acid, keeping the vaginal pH under 4.5 and thus preventing the growth of pathogenic or opportunistic micro-organisms such as *Candida* spp. or *Gardnerella* spp. [4]. However, this estrogenic tone, which should be a protective factor in patients with PCOS, is only basal, and the necessary increases associated with the normal menstrual cycle do not occur [5]; thereby leadings to increased destruction of epithelial cells and local inflammation [7]. These conditions create a favorable environment for microorganisms to colonize the lower genital tract. *Gardnerella vaginalis*, *Prevotella* spp., and *Mycoplasma hominis* have been described with a higher prevalence in the vaginal microbiota of patients with PCOS [8]. This disruption of the vaginal microbiome would potentially lead to an increase in local inflammatory factors, such as interleukin-8 and tumor necrosis factor-α, which, together with other metabolites, trigger chronic systemic inflammation that can affect the hypothalamic–pituitary–ovarian axis. At the vaginal level, the increase in polymorphonuclear neutrophils (PMN), and their activation induces the formation and release of neutrophil extracellular traps (NETs), which allow PMN to perform a process of cell death (NETosis) through which the release of nuclear chromatin, peptides, and antimicrobial enzymes, including defensins, catalysins, neutrophil elastase (NE) and myeloperoxidase (MPO) occurs, controlling and helping the organism to defend itself from inflammatory and infectious processes [9,10,11,12].

The effect of sex steroid hormones as well as the intestinal microbiome have been extensively investigated [13,14,15], but inflammatory states and the vaginal microbiota require further evaluation [16,17]. This study explores the diversity of the vaginal microbiota in patients with PCOS, as well as the vaginal inflammatory reaction (VIR) and PMN activation with the production of neutrophil extracellular traps.

## 2. Materials and Methods

### 2.1. Study Design

Cross-sectional study.

### 2.2. Sample

The sample corresponded to the first 33 patients who attended the consultation at the Hospital UTPL-Santa Inés de Loja, Ecuador, from May to August 2023 and diagnosed with PCOS. This study was considered random due to the characteristics of the sampling.

### 2.3. Location

Conducted in 2022–2023 at the Hospital Santa Inés, Loja, Ecuador, and the Center of Excellence in Translational Medicine and Scientific and Technological Bioresource Nucleus (CEMT-BIOREN), Universidad of La Frontera, Temuco, Chile.

### 2.4. Ethical Aspects

The women participating in the study participated voluntarily and without monetary reward. They were given a written informed consent form that was read and explained to them, and a copy was provided and signed by each of them. The Human Research Ethics Committee (CEISH) 2023-006O-IE of the University of Cuenca, Ecuador, authorized the research protocol.

### 2.5. Survey Application

A survey was applied anonymously (using a code), which allowed the collection of clinical data of women with a diagnosis of PCOS. The items included in the survey were the following: age, age of onset of sexual intercourse, number of sexual partners in the last six months, use of oral contraceptives, oligomenorrhea. At the same time, blood tests for the following hormones were performed: HOMA (Homeostasis Model Assessment), insulin, total testosterone, DHEAS (dehydroepiandrosterone sulfate), 17—OH progesterone, androstenedione (A4), LH (luteinizing hormone), FSH (follicle stimulating hormone), AMH (Anti-Müllerian hormone), glucose, and vitamin D.

### 2.6. Patients

Thirty-three women over 18 years of age diagnosed with PCOS were included according to the recommendations of the 2018 international evidence-based guidelines [2], which allow for the classification of PCOS patients according to phenotypes A, B, C, and D. Exclusion criteria were as follows: menstruation on the day of sampling, having had sexual intercourse in the last 48 h, being a douche user, cognitive impairment, antimicrobial treatment in the last 30 days, immunosuppressive drugs or immunosuppression, chronic degenerative diseases (non-classical congenital adrenal hyperplasia, androgen-producing tumor, hyperprolactinemia, thyroid dysfunction, Cushing’s syndrome, drugs with androgenic activity, etc.), and patients with ovarian cysts.

Four clinical phenotypes of the disease have been identified, each with clinical implications regarding severity. Phenotype A is known as “classic” or complete and consists of three criteria: hyperandrogenism, oligo-ovulation, and polycystic ovarian morphology. Phenotype B, also called “classic”, has hyperandrogenism and oligo-ovulation. Both phenotypes A and B have a more severe clinical and metabolic impact. Phenotype C is called “ovulatory”, characterized by hyperandrogenism and polycystic ovarian morphology, while phenotype D, “non-hyperandrogenic”, is composed of oligo-ovulation and polycystic ovarian morphology, and being less severe [18].

### 2.7. Vaginal Discharge Samples

The samples were taken through speculoscopy by a professional gynecologist, using a cotton swab from the “vaginal fornix and vaginal walls”. Subsequently, 5 smears were made on slides which were left to dry in the air and then stored in a transporting box. Each slide was used for the following purposes: DNA extraction, Gram staining, Giemsa, and immunofluorescence.

#### 2.7.1. DNA Extraction

Vaginal material was scraped with a scalpel from each slide and placed in 1.5 mL tubes with lysis buffer (0.01 M Tris pH 7.8, 0.005 M EDTA pH 8, 0.5% SDS) and incubated at 85 °C for 15 min. Subsequently, 7.5 M ammonium acetate pH 7.5 was added, the tubes were cooled to −20 °C for 5 min, and the proteins were precipitated at 12,000 rpm for 5 min (min) at 4 °C. The supernatant was mixed with isopropanol and incubated overnight at −20 °C. DNA was precipitated at 12,000 rpm for 15 min at 4 °C, washed with 70% ethanol, and hydrated with TE buffer (10 mmol/L Tris-HCl, 1 mmol/L EDTA pH 8). Internal control: a conventional one-step polymerase chain reaction (PCR) was used as an internal control, which allowed us to discard samples with non-amplifiable inhibitory DNA. For this, primers PCO4 and GH20 were used: primers PCO4 5′-caacttcatccacgttcacc-3′ and GH20 5′-gaagagccaaggacaggacaggacaggtac-3′, which amplify a fragment of 268 base pairs (bp) of the Beta-Globin gene.

#### 2.7.2. Microorganism DNA Detection

Conventional one-step PCRs were used for *Chlamydia trachomatis*, *Candida albicans* [19], and *Trichomonas vaginalis* [20], using specific primers previously published. For the detection of *Gardnerella vaginalis* [21], *Atopobium vaginae*, and *Megasphaera phylotype* 1 [22], a conventional one-step multiplex PCR was used. Primer sequences and fragment sizes are shown in (Table 1). Positive controls: cultures or known clinical samples positive for each microorganism were used. Visualization: PCR products were confirmed relative to the positive control on 1.6% agarose gels stained with GelRed using a 100 bp marker (New England Biolabs, Ipswich, MA, USA).

**Figure 1:**
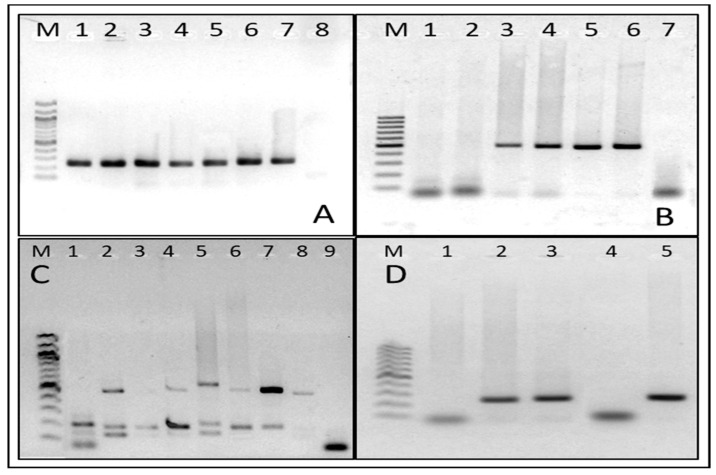
**Representative images of PCR products from the conventional PCRs used in the study:** (**M**) Molecular weight marker (100 bp) (**A**) PCR of the Beta-globin (268 bp) gene used as an internal control; (**B**) PCR of *Candida albicans* (496 bp), where lanes 1 and 2 are negative samples, lanes 3, 4, and 5 are positive samples, lane 6 is positive control, and lane 7 is blank control; (**C**) multiplex PCR for *Gardnerella vaginalis* (206 bp), *Atopobium vaginae* (558 bp) and *Megasphaera phylotype* 1 (144 bp), where lanes 1 and 2 are positive controls, lanes 3, 4, 5, 6, 7, and 8 are positive samples, and lane 9 is blank control; and (**D**) *Chlamydia trachomatis* PCR (241 bp), where lane 1 is negative sample, lanes 2 and 3 are positive samples, lane 4 is blank control, and lane 5 is positive control.

The result was determined by visualization of a band at the same height, as the positive control was interpreted as a positive sample for that micro-organism. Conversely, no band visualization was interpreted as a negative result (Figure 1).

### 2.8. Analysis of Vaginal Discharge Smears

The analysis of the smears was carried out according to the criteria stated in the BACOVA-ERIGE (Balance of Vaginal Contents-Study of the Genital Inflammatory Reaction) procedures manual, which classifies the vaginal microbiota into 5 basic vaginal states [24] (Table 2).

#### 2.8.1. Gram Stain

Microscopic observation using a 100× objective and immersion oil allows differentiation between Gram-positive bacteria (violet color) and Gram-negative bacteria (reddish color). The reading was performed in 20 fields, counting Gram-positive bacilli (*Lactobacillus* spp.), pleomorphic Gram-variable bacilli (compatible with *Gardnerella vaginalis*), and Gram-variable curved bacilli (*Mobiluncus* spp.). A score was assigned to the number of morphotypes found, according to Nugent’s criteria [25], allowing the microbiota to be classified with the following score: normal (NM) 0–3, intermediate (IM) 4–6, and bacterial vaginosis (BV) 7–10. In addition, the presence or absence of blastoconidia and pseudohyphae were recorded.

#### 2.8.2. Proinflammatory State

Observed by Gram staining and defined by the amount of cellular damage (referred to as destroyed cells (detritus) and/or epithelial cell nuclei without cytoplasm), the following criteria were assigned: absent (0); present, grouping the cases that presented scarce (1+); moderate (2+); and intense (3+) cellular damage. The evaluation was performed in 20 fields at 1000× with immersion oil.

#### 2.8.3. Vaginal Inflammatory Reaction (VIR)

VIR was observed by Giemsa staining and counting leukocytes or PMN, applying criteria according to the Bacova–Erige manual [24]. Absent VIR: <5, moderate VIR: >5–<10, and intense VIR: >10 PMN per field. Reading was performed in 5 non-adjacent fields with a 100× objective and immersion oil.

#### 2.8.4. Interpretation of Results

##### Nonspecific Microbial Vaginitis (nMVitis)

All vaginal discharge smears that had a Nugent score between 4 and 10 and presented VIR (moderate or intense) were classified in this category. Vaginal microbiota with these characteristics were considered to present a proinflammatory state or acute inflammation.

##### Bacterial Vaginosis

Interpreted as a state of chronic inflammation of the vaginal microbiota, since an inflammatory reaction with the presence of PMN was not observed.

##### Definition of Altered and Normal Microbiota

For the preparation of the tables and statistical analysis of the group under study, all cases with intermediate microbiota, bacterial vaginosis, and cases with nMVitis were considered altered microbiota. According to Nugent’s criteria, cases that scored between 0 and 3 were considered normal microbiota.

#### 2.8.5. Identification of Neutrophil Extracellular Traps (NETs)

Immunofluorescence of vaginal discharge smears was performed to determine the presence of neutrophil extracellular DNA using a polyclonal primary antibody anti-rabbit IgG (reference no. ab68672 Abcam, Cambridge, UK) to identify NE elastase. The smears were incubated with polyclonal anti-NE antibody (reference no. ab68672 Abcam, Cambridge, UK) in PBS-BSA at a dilution of 1:300. They were then washed by manual shaking and incubated for 1 h with secondary antibody anti-Rabbit IgG conjugated with Alexa Fluor™ 488 (reference no. A11008, Life Technologies, Invitrogen, Thermo Fisher Scientific, Waltham, MA, USA). Subsequently, the samples were then washed by manual shaking with Sytox orange^®^ (reference no. s11368, Invitrogen, Thermo Fisher Scientific, Waltham, MA, USA) (stain for DNA, 1:2000 in 1X PBS). The slides were covered with mounting medium (reference no. 00-4959-52, Invitrogen Thermo Fisher Scientific, Waltham, MA, USA) and a coverslip. The TissueFAXS i Plus Cytometry microscope (TissueGnostics, Vienna, Austria) was used to scan the smears. Then, the TissueFaxs-Viewer 7.0 software was used to obtain images of interest areas to observe fluorescence.

### 2.9. Statistical Analysis

The data obtained were entered in an Excel spreadsheet; they were analyzed with the SPSS program (IMB-SPSS, version 29.0 for Windows); descriptive statistics were used with the calculation of frequencies, percentages, measures of central tendency and dispersion, inferential statistics, and parametric and nonparametric tests according to each variable. For clinical and hormonal variables, OR values were calculated with their respective 95% CI. Values of *p* ≤ 0.005 were considered statistically significant.

## 3. Results

The participants’ age range was 18–36 (mean 23 years). Phenotype A was the most representative, with 60.6% (20/33), C and D both had 18.2% (6/33), and B was the least observed (1/33) 3.0%.

Vaginal microbiota status according to Bacova–Erige criteria were NM 30.3% (10/33), IM 33.3% (11/33), BV 15.2% (5/33), and nonspecific microbial vaginitis (nMVitis) 21.2% (7/33). A high number of patients, (23/33) 69.7%, presented an altered microbiota.

The variables age (≤23 years *p*-value = 0.07), phenotypes A and B (considered together), lifetime sexual partners, and oligomenorrhea represented the highest number of women with altered microbiota. Oral contraceptive use was the variable with the slightest variation among users and non-users of contraceptives. However, the most important and significant statistical association was observed with the variable active sexually transmitted infection at the time of sampling (*p*-value = 0.023) (Table 3).

About the hormonal variables, only insulin showed a significant *p*-value. More cases were observed in patients with normal insulin and altered microbiota. Glucose levels were also analyzed in association with the state of the microbiota, but no results could be obtained since the glucose levels of all the patients were reported as normal (Table 4).

The proinflammatory state assessed by cell destruction (debris) and naked nuclei was present in 72.7% (24/33) of cases with variable intensity (scarce 6/33, moderate 10/33, and intense 8/33), depending on whether they were found in some or all fields observed in Gram staining. A statistically significant difference was observed between the proinflammatory statuses present vs. absent concerning normal or altered vaginal microbiota *p*-value = 0.021. A higher percentage of proinflammatory status and altered vaginal microbiome was observed in 14 (60.9%) patients (Table 5).

VIR was evaluated by the presence of PMN and Giemsa staining, and was present in 24.2% (8/33, 1 NM case and 7 IM cases).

The release of NETs was observed in the eight cases that presented VIR, and their characteristics are shown in Table 6. In cases with VIR, 62.5% (5/8) are of phenotype A, with no cases of phenotype B found. A total of 25% (2/8) of phenotype D and 12.5% (1/8) of phenotype C were also found. The proinflammatory status of the cases analyzed for NETs were as follows: 50% (4/8) intense, 25% (2/8) moderate, 12.5% (1/8) scarce, and 12.5% (1/8) absent. Moreover, regarding the vaginal microbiota, 87.5% (7/8) of the cases presented nMVitis.

By PCR, one-eighth of the samples were positive for *Candida albicans*, two-eighths were positive for *Atopobium vaginae*, and seven-eighths were positive for *Gardnerella vaginalis*. For *Trichomonas vaginalis* and *Chlamydia trachomatis*, all were negative.

**Figure 2:**
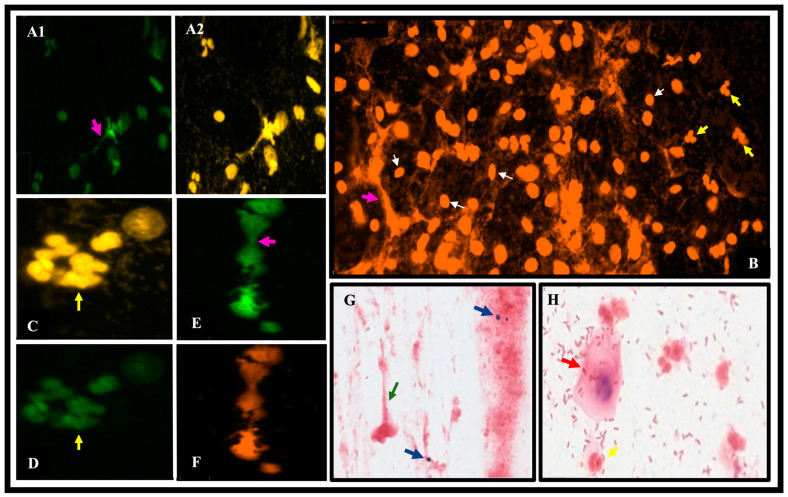
Representative images of vaginal discharge smears from women with PCOS. The TissueFAXS i Plus cytometry microscope was used to scan the smears at 20X magnification and TissueFaxs-Viewer software was used to obtain images at 100-450% zoom. Green staining in (**A1**,**D**,**E**) shows anti-NE labeling (Alexa fluor 488); Sytox orange staining marks DNA in images (**B**,**F**); Gram staining (**G**) shows structures suggestive of NET (green arrow), and presence of blastoconidia corresponding to Candida albicans diagnosed by PCR (blue arrows); Gram-stained image (**H**) indicates vaginal squamous cell (red arrow) showing different bacterial morphotypes corresponding to an intermediate microbiota; image (**F**) marks PMNs in the process of DNA release; yellow arrows indicate PMN nuclei (**B**,**C**,**D**,**H**); white arrows indicate squamous epithelial cell nuclei (**B**) and fuchsia arrows indicate NETs (**A1**,**E**) and (**A2**,**B**) channel merging. Images (**C**,**D**,**E**,**F**) (450% zoom), (**A1**,**A2**,**G**,**H**) (230% zoom), and (**B**) (100% zoom) were used.

As observed in (Figure 2) representative images of vaginal discharge smears from women with PCOS show anti-NE marking (Alexa fluor 488), sytox orange staining marking DNA, and Gram stain-marked structure suggestive of NETs (green arrow).

The PCR results for each of the samples analyzed are shown in Table 7. In Gram staining, yeast (blastospores) were observed in 6/33 cases; four were positive for *Candida albicans* by PCR, and two were positive for non- C. *albicans*. No positive cases were detected for *Trichomonas vaginalis.*

## 4. Discussion

The presence of an altered microbiota was a frequent condition in the studied PCOS patients (69.7%), with a high prevalence of non-specific microbial vaginitis (21.2%). A significant statistical association (*p*-value = 0.023) was observed between the variable active STI and VM (Table 3). On the other hand, the proinflammatory state, defined as evidence of cell destruction and loose nuclei, had a high frequency (72.7%). Additionally, the VIR was present in 8/33 of PCOS patients with PMN counts greater than five per field, with the presence of NETs observed in all cases.

In this scenario of altered vaginal microbiota, it could be a particular and frequent event in women diagnosed with PCOS, where hormonal and metabolic disorders they present could play a role in NETs release [26]. Changes in the composition of the vaginal microbiota could be associated with metabolic alterations, including insulin resistance, suggesting a link between metabolic health and the vaginal microbiota. However, in this study, altered blood insulin levels were not found, and 86.7% of the patients presented normal insulin values.

Estrogens are related to the production of glycogen, which is the substrate necessary for *Lactobacilli* to grow, produce lactic acid, and maintain a low pH, which contributes to a pathogen-free vaginal environment. Elevated estrogen states, as seen during puberty and pregnancy, promote the preservation of a homeostatic (eubiotic) vaginal microenvironment by stimulating the maturation and proliferation of vaginal epithelial cells and the accumulation of glycogen [7]. However, the relationship between 17-B estradiol and Lactobacilli is not entirely understood within the context of the pathophysiology of PCOS [27,28]. In our study, the intermediate microbiota condition was the most frequent, at 33.3% in patients with PCOS. It is precisely in this condition where we begin to observe a VM with some degrees of alteration: lower presence of *Lactobacillus* and a higher presence of bacterial morphotypes of *Gardnerella vaginalis*, *Atopobium vaginae* type, and some others that usually do not cohabit in the vaginal tract. The patients in the present study presented a high frequency of *Gardnerella vaginalis* (66.7%), observed by PCR.

Regarding the epithelial damage (defined as proinflammatory state) associated with PCOS patients in the Gram reading, in over 70% of the smears, destroyed cells and naked nuclei were visualized. In cases with normal microbiota and nonspecific microbial vaginitis, 100% exhibited these characteristics with varying degrees of intensity. A similar condition is found in cytolytic vaginosis (CV), characterized by fragmented epithelial cells and abundant Lactobacilli, which causes an increase in vaginal acidity and epithelial damage [29]. These laboratory findings could help distinguish between CV and candidiasis, as these pathologies do not differ in clinical features [16]. Despite this, there is still a lack of knowledge about CV and its scope [29]. These processes associated with the pathophysiology of inflammation increase the presence of PMN-generated NETs in the vaginal discharge of women with fungal, bacterial, and parasitic infections, indicating the development of infectious foci at the vaginal level [30]. This study used identifying NETs as an indicator of activated PMNs [30,31]. Our observations showed the presence of NETs in all studied cases presenting with a VIR, which had normal microbiota (one-eighth) and non-specific microbial vaginitis (seven-eighth), as shown in Table 6. By these results, we could speculate a scenario that this syndrome by itself could trigger a proinflammatory state at the level of the vaginal epithelium, independent of the state of the vaginal microbiota.

These observations are consistent with those reported by Jin C et al. (2023) in their case-control study, where they found a higher degree of heterogeneity in the vaginal microbiome. As well as the association with other vaginal infections, *Gardnerella vaginalis* was observed more frequently (66.7%) in all stages of VM, whether accompanied by *Atopobium vaginae*, *Megasphaera phylotype* 1, or yeasts such as *Candida albicans* and a higher frequency of bacteria such as *Gardnerella vaginalis* compared to the control group [5]. Another study demonstrated a higher incidence of vaginitis in women with PCOS compared to the control group [32].

Additionally, this is the first study that shows evidence of NETotic activity at the level of the vaginal epithelium in women with PCOS. This condition, associated with amenorrhea or oligomenorrhea states, could contribute to sperm entrapment by NETs generated by activated PMNs, which could increase the risk of infertility in these patients.

## 5. Conclusions

In the studied series, more women with altered microbiota were noted in clinical variables, phenotypes A and B, sexual partners (>2), and oligomenorrhea. However, only the active STI and insulin variables showed a significant *p*-value. This is the first study to demonstrate the presence of NETs in eight cases, showing NETotic activity in women with PCOS. We also highlight the high frequency of a proinflammatory state observed across all vaginal microbiota states. These findings suggest that the syndrome itself could trigger a proinflammatory state in the vaginal epithelium, independent of the vaginal microbiota’s state. Additionally, these conditions, associated with amenorrhea or oligomenorrhea, could lead to decreased fertility in these PCOS patients.

**Limitations of the study**. The limitations include the lack of an average population (control group) for comparisons between VM, VIR, proinflammatory status, and other variables. However, despite a small n, trends were observed when calculating the *p*-value. We hope that this study will serve as a basis for future studies in women with PCOS, including a more significant number of participants both with PCOS and a control group (without PCOS) in order to generate additional data to contribute to a better understanding of this pathology.

## Figures and Tables

**Table 1:** Primers used in PCR analysis.

Micro-Organism	Primers	Sequence 5′-3′	Fragment Base Pairs	Reference	GenBank
*Chlamydia trachomatis*	Forward	tccggagcgagttacgaaga	241	Sánchez V. 2009 [23]	NZ_CP009926.1
	Reverse	aatcaatgcccgggattggt			
*Candida albicans*	Forward	atgggtggtcaacatac tacatctatgtctaccacc	496	Burgener-kairuz 1994 [19]	X13296.1
	Reverse				
*Trichomonas vaginalis*	Forward	attgtcgaacattggtcttaccctc	262	Pačes J, 1992 [20]	L23861.1
	Reverse	tctgtgccgtcttcaagtatgc			
*Gardnerella vaginalis*	Forward	gcgggctagagtgca acccgtggaatgggcc	206	Zariffard MR, 2002 [21]	GenBank: AY738665.1
	Reverse				
*Atopobium vaginae*	Forward	gcagggacgaggccgcaa	558	Fredricks D. 2007 [22]	AY738657.1
	Reverse	gtgtttccactgcttcacctaa			
*Megasphaera phylotype* 1	Forward	gatgccaacagtatccgtccg	144	Fredricks D. 2007 [22]	AY738672.1
	Reverse	Acagacttaccgaaccgcct			

**Table 2:** Definition of basic vaginal states.

**BVS in Women’s Childbearing Age**	NV	VIR
**I Normal microbiota** Predominance of *Lactobacilli*	0–3	No
**II Normal microbiota + VIR** Predominance of *Lactobacilli* with vaginal inflammatory reaction present.	0–3	Yes
**III Intermediate microbiota** Equilibrium of *Lactobacilli* and anaerobic Bacteria	4–6	No
**IV Bacterial vaginosis** Predominance of anaerobic bacteria	7–10	No
**V Nonspecific Microbial Vaginitis** Alteration of the ratio of *Lactobacilli* and anaerobic bacteria, or presence of foreign bacterial morphotypes with inflammatory reaction.	4–10	Yes

**BVS**: basic vaginal status; **NV**: Nugent’s numerical value, **VIR**: vaginal inflammatory reaction.

**Table 3:** Clinical factors according to vaginal microbiota status in the study group (n = 33).

		Vaginal Microbiota (%)			
Variables	n	Normal	Altered	OR (IC)	*p*-Value
**Phenotypes**				2.29(0.50; 10.50)	0.283
A and B	21(63.6)	5(23.8)	16(76.2)		
C and D	12(36.4)	5(41.7)	7(58.3)		
**Age of onset of** **sexual intercourse (years)**				0.27(0.04; 1.57)	0.133
≤18	20(60.6)	8(40.0)	12(60.0)		
>18	13(39.4)	2(15.4)	11(84.6)		
Number of sexual partners				0.35(0.06; 2.15)	0.246
None	6(18.18)	3(50.0)	3(50.0)		
More than 1 sexual partner	27(81.82)	7(25.93)	20(74.07)		
**Active STI**				6.68(1.18; 37.78)	* 0.023
Yes	25(75.8)	5(20.0)	20(80.0)		
No	8(24.2)	5(62.5)	3(37.5)		
**Oral contraceptives use**				0.47(0.10; 2.27)	0.341
Yes	19(57.6)	7(36.8)	12(63.2)		
No	14(42.4)	3(21.4)	11(78.5)		
**Oligomenorrhea**				2.86(0.46; 17.58)	0.246
Yes	27(81.8)	7(25.9)	20(74.1)		
No	6(18.2)	3(50.0)	3(50.0)		

**Number of sexual partners**: in the last six months. **Active STI**: sexually transmitted infections detected in the laboratory by conventional PCR. **Statistical test:** Chi-squared, **OR**: Odds Ratio, **IC**: confidence interval, * *p*-value < 0.005.

**Table 4:** Hormonal variables according to vaginal microbiota status in the study group (n = 33).

		Vaginal Microbiota (%)			
Hormonal Variables	N	Normal	Altered	OR (IC)	*p*-Value
**HOMA**				0.52(0.10; 2.60)	0.429
Normal	12(40.0)	5(25.0)	9(75.0)		
Altered	18(60.0)	7(38.9)	7(61.1)		
Insulin				_	0.002
Normal	26(86.7)	6(23.1)	20(76.9)		
Altered	4(15.3)	4(100.0)	_		
**Total Testosterone**				0.28(0.06; 0.35)	0.103
Normal	16(48.5)	7(43.8)	9(56.3)		
Altered	17(51.5)	3(17.6)	14(82.4)		
**DHEAS**				_	0.220
Normal	26(89.7)	9(34.6)	17(65.4)		
Altered	3(10.3)	-	3(100.0)		
**17-OH progesterone**				2.22(0.43; 11.6)	0.339
Normal	15(53.6)	6(40.0)	9(60.0)		
Altered	13(46.4)	3(23.1)	10(76.9)		
**Androstenedione**				1.07(0.2; 5.7)	0.930
Normal	20(69.0)	7(35.0)	13(65.0)		
Altered	9(31.0)	3(33.3)	6(66.7)		
**LH**				1.36(0.29; 6.28)	0.690
Normal	17(54,8)	6(35.3)	11(64.7)		
Altered	14(45.2)	4(28.6)	10(71.4)		
**AMH**				2.42(0.13; 44.50)	0.540
Normal	2(7.7)	1(50.0)	1(50.0)		
Altered	24(92.3)	7(29.2)	17(70.8)		
**Vitamin D**				0.23(0.03; 2.61)	0.234
Normal	20(71.4)	7(35.0)	13(65.0)		
Altered	8(28.6)	1(12.5)	7(87.5)		

**DHEAS**: dehydroepiandrosterone sulfate; **LH**: luteinizing hormone; **AMH**: Anti-Müllerian hormone. **Statistical test:** Chi-squared, **OR**: Odds Ratio, **IC**: confidence interval, *p*-value < 0.005.

**Table 5:** Proinflammatory state according to the states of the vaginal microbiota.

	Proinflammatory State (%)			
Vaginal Microbiota	n (%)	Absent	Present	*p*-Value
Normal	10(30.3)	0	10(100.0)	* 0.021
Altered	23(33.3)	9(39.1)	14(60.9)	
**Total**	**33(100.0)**	**9(27.3)**	**24(72.7)**	

The absence or presence of a proinflammatory state was defined by the amount of cell destruction and loose nuclei observed during Gram reading in 20 fields. Vaginal microbiota normal: a score between 0 and 3 was considered according to the Nugent criteria. Altered vaginal microbiota includes intermediate microbiota (IM), bacterial vaginosis (BV), and non-specific microbial vaginitis (nMVitis). **Statistical test:** Chi-squared, (*) corresponds to a statistically significant *p*-value (*p*< 0.05).

**Table 6:** Distribution of SOP phenotype, proinflammatory status, and vaginal microbiota in the eight cases examined for neutrophil extracellular traps (NETs).

Cases with VIR	Phenotypes	Proinflammatory State	Vaginal Microbiota
1	D	Scarce	MN
2	A	Intense	nMVitis
3	D	Intense	nMVitis
4	A	Moderate	nMVitis
5	A	Moderate	nMVitis
6	C	Intense	nMVitis
7	A	Absent	nMVitis
8	A	Intense	nMVitis

**(VIR):** vaginal inflammatory reaction; **(nMVitis):** non-specific microbial vaginitis.

**Table 7:** Micro-organisms detected by PCR in vaginal discharge samples (n = 33).

Micro-Organisms	PCR Results	
	Positive	Negative
*Gardnerella vaginalis*	22(66.7)	11(33.3)
*Atopobium vaginae*	10(30.3)	23(69.7)
*Megasphaera type* 1	4(12.1)	29(87.9)
*Trichomonas vaginalis*	0	0
*Chlamydia trachomatis*	2(6.1)	31(93.9)
*Candida albicans*	4(12.1)	29(87.9)

## Data Availability

The data supporting the findings of this study are available from the corresponding author upon reasonable request.
